# Effects of a dual CCR3 and H_1_-antagonist on symptoms and eosinophilic inflammation in allergic rhinitis

**DOI:** 10.1186/1465-9921-11-17

**Published:** 2010-02-09

**Authors:** Lennart Greiff, Cecilia Ahlström-Emanuelsson, Ash Bahl, Thomas Bengtsson, Kerstin Dahlström, Jonas Erjefält, Henrik Widegren, Morgan Andersson

**Affiliations:** 1 Department of ORL, Head & Neck Surgery, Lund University Hospital, Lund, Sweden; 2 New Opportunities, AstraZeneca R&D, Charnwood, UK; 3 Clinical Information Science, AstraZeneca R&D, Lund, Sweden; 4 Department of Experimental Medical Science, Lund University, Lund, Sweden

## Abstract

**Background:**

The CC-chemokine receptor-3 (CCR3) has emerged as a target molecule for pharmacological intervention in allergic inflammation.

**Objective:**

To examine whether a dual CCR3 and H_1_-receptor antagonist (AZD3778) affects allergic inflammation and symptoms in allergic rhinitis.

**Methods:**

Patients with seasonal allergic rhinitis were subjected to three seven days' allergen challenge series. Treatment with AZD3778 was given in a placebo and antihistamine-controlled design. Symptoms and nasal peak inspiratory flow (PIF) were monitored in the morning, ten minutes post challenge, and in the evening. Nasal lavages were carried out at the end of each challenge series and α_2_-macroglobulin, ECP, and tryptase were monitored as indices of allergic inflammation.

**Results:**

Plasma levels of AZD3778 were stable throughout the treatment series. AZD3778 and the antihistamine (loratadine) reduced rhinitis symptoms recorded ten minutes post challenge during this period. AZD3778, but not the anti-histamine, also improved nasal PIF ten minutes post challenge. Furthermore, scores for morning and evening nasal symptoms from the last five days of the allergen challenge series showed statistically significant reductions for AZD3778, but not for loratadine. ECP was reduced by AZD3778, but not by loratadine.

**Conclusions:**

AZD3778 exerts anti-eosinophil and symptom-reducing effects in allergic rhinitis and part of this effect can likely be attributed to CCR3-antagonism. The present data are of interest with regard to the potential use of AZD3778 in allergic rhinitis and to the relative importance of eosinophil actions to the symptomatology of allergic rhinitis.

**Trial registration:**

EudraCT No: 2005-002805-21.

## Background

The CC-chemokine receptor-3 (CCR3) is a transmembrane protein that constitutes one of the receptors for CC-chemokines. It is localized to cells of key importance to allergic inflammation including dendritic cells, Th2-lymphocytes, eosinophils, basophils, and mast cells as well as to epithelial, smooth muscle, and neural cells [1-7]. Chemokines interacting with CCR3 include eotaxin-1, 2, and 3, MCP-4, and RANTES [8]. Stimulation of the receptor produces chemotaxis and cellular activation [8], and experiments in CCR3 knock-out mice and such involving CCR3-neutralizing antibodies have demonstrated the importance of the CCR3-pathway to eosinophil activity *in vivo *[9-12]. CC-chemokines are increased in allergic airway conditions [10,13-16]. Accordingly, CCR3 may be a treatment target in allergic rhinitis and asthma. Recently, this possibility was substantiated by a report on symptom-reducing effects of topical anti-sense therapy directed towards CCR3 in asthma [17], although that particular study did not discriminate between an effect on CCR3 and an effect mediated through the common β-chain of the IL-3, IL-5, and GM-CSF receptors.

A series of experimental studies have shown that small molecular weight CCR3-antagonists can reduce allergic inflammation *in vivo*. Wegmann *et al*., in a study involving ovalbumin-sensitized mice repeatedly challenged with ovalbumin to produce inflammation, showed that a CCR3-antagonist reduced BAL and tissue eosinophila and that this effect was associated with normalization of airway reactivity and prevention of goblet cell hyperplasia [18]. Das *et al*. reported that two separate CCR3-antagonists reduced eotaxin-elicited and allergen-induced eosinophil recruitment to bronchial airways in an experimental murine model [19]. Nakamura *et al*., in a mouse model of allergic conjunctivitis, demonstrated that a highly selective CCR3-antagonist attenuated early and late phase symptoms [20], suggesting a symptom-reducing potential in allergic conditions, and that the effect was associated with mast cell stabilization. Additional observations on effects of CCR3-antagonists comprise prevention of immediate and late-phase allergic skin reactions to allergen in a mouse allergy model [21], inhibition of eosinophil infiltration into the airways of monkeys following segmental bronchial provocation with eotaxin [22], and dose-dependent reduction of eosinophil recruitment into the lungs in an animal model of allergic airway inflammation [23]. The examples above highlight the anti-allergic potential of CCR3-antagonism. However, observations on effects of CCR3-antagonists on allergic inflammation in man are lacking. Hence, explorations of CCR3-inhibition in man are highly warranted.

AZD3778 is a novel small molecular weight dual CCR3 and histamine H_1_-receptor antagonist. It has been characterized by MDS Pharma Services (Taipei, Taiwan: http://discovery.mdsps.com) in a battery of 226 assays covering a diverse range of enzyme, transporter, and receptor targets (not including CCR3). The only significant activity detected (defined as >50% inhibition) was against the guinea-pig H_1_-receptor with a Ki of 54 nM. AZD3778 also has been shown to inhibit the binding of a specific CCR3 radioligand, ^125^I-eotaxin, to the human CCR3-receptor expressed on CHO-cells with a pIC50 of 8.1 ± 0.1 (mean ± SEM, equivalent to an IC50 of 8 nM.) The functional potency of AZD3778 has been assessed in test systems in which a response mediated by human CCR3 could be evoked using human eotaxin 1 or 2. For example, AZD3778 inhibited chemotaxis of eosinophils. From *in vitro *experiments on whole blood, where plasma protein binding would reduce the free fraction of AZD3778, the A_2 _(the concentration of antagonist required to produce a two-fold shift of the agonist response) for CCR3 was 200 nM. AZD3778 also inhibited the binding of a specific H_1_-radioligand, ^3^H-pyrilamin, to the human H_1_-receptor expressed on CHO-cells with an IC_50 _of 40 nM. In human HeLa-cells expressing the human H_1_-receptor, AZD3778 inhibited histamine-induced calcium flux with an IC_50 _of 63 nM (expected A_2 _in whole blood of 1000 nM). Taken together, the above observations indicate that AZD3778 has CCR3 and H_1_-antagonistic properties.

In the present study, involving patients with allergic rhinitis examined outside the pollen season in an allergen challenge model [24,25], we examined whether treatment with AZD3778 affected symptoms and signs of allergic rhinitis. Accordingly, we monitored rhinitis symptoms and nasal lavage fluid levels of ECP, reflecting eosinophil activity, as well as tryptase and α_2_-macroglobulin, reflecting mast cell activity and plasma exudation, respectively. We have previously shown that nasal lavage recovery of inflammatory mediators can be improved by utilization of histamine-challenges. In this regard, histamine produces a plasma exudation response that facilitates their luminal entry [25]. In the present study, we aimed at using this experimental tool, but since AZD3778 exerts H_1_-antagonistic effects we chose to induce plasma exudation through nasal challenge with bradykinin [26]. Finally, reflecting the H_1_-antagonistic property of AZD3778, we compared the effect of this drug to an anti-histamine (loratadine). We report on symptom-reducing and anti-eosinophil effects that likely can be attributed to CCR3-antagonism.

## Methods

### Study design

The study was of a randomized, double blind, placebo-controlled (double-dummy), and three-way crossover design. It comprised three ten days' treatment periods carried out in the pollen-free autumn/winter months separated by at least two weeks washout-periods. The treatments were AZD3778, loratadine, and placebo. After three days of treatment, a series of seven individualized, once-daily allergen challenges commenced while the treatment continued. Nasal symptoms and peak inspiratory flow (PIF) were recorded. In addition, at the end of each treatment and allergen challenge series, nasal lavages were carried out. Tryptase, ECP, and α_2_-macroglobulin were measured as indices of mast cell activity, eosinophil activity, and plasma exudation, respectively. The study was approved by the Regional Ethics Committee, Lund, and the Swedish Medical Product Agency. It was conducted according to the Declaration of Helsinki and in compliance with Good Clinical Practice. Informed consent was obtained.

### Subjects

Forty-six patients were enrolled and subjected to physical examination and skin prick test. Inclusion criteria: Men and post-menopausal or surgically sterile women, seasonal allergic rhinitis for at least two years, positive skin prick test to birch or grass pollen allergen, asymptomatic condition outside the pollen season, need for treatment at seasonal allergen exposure, and a positive response to nasal allergen challenge (see the titration procedure below). Exclusion criteria: Any relevant disease including perennial allergic rhinitis, asthma, clinically relevant structural nasal abnormalities, upper respiratory tract infection within two weeks prior to the start of the study, use of topical corticosteroids within four weeks prior to the study and use of antihistamines within one week, and immunotherapy. Of the 46 subjects enrolled, 38 were randomized and received at least one dose of a study drug. Of those randomized, 37 were analyzed for efficacy. There were six withdrawals from the study: One due to an adverse event and five because of lack of compliance. Of the 38 subject allocated to treatment, all were men. The average age was 25 years (range 20-51) and average BMI was 24 kg/m^2 ^(range 20-29). The median time since diagnosis of allergic rhinitis was 14 years (range 2-33).

### Treatment

AZD3778 (AstraZeneca, Lund, Sweden) was administered as an oral suspension (300 mg twice daily). Loratadin Biochemie (Sandoz, Helsingborg, Sweden) was given as a tablet (10 mg once daily). Placebo was a suspension to match AZD3778 (AstraZeneca, Lund, Sweden) and a tablet to match Loratadin Biochemie (AstraZeneca, Charnwood, UK). Each treatment period comprised ten days and compliance was assured by supervising administration on the first treatment day and thereafter by control of daily diary recordings of intake of study medication. Allergen challenges (see below) were administered during the last seven days of each treatment period. On the first day of treatment as well as on days eight, nine, and ten, pharmacokinetic samples (plasma) were collected within two to five hours after drug administration. Also, such samples were obtained before drug administration on days eight, nine, and ten.

### Allergen challenge model

In order to establish individually tolerable, repeatable, yet symptom-producing allergen challenge-doses, a nasal titration procedure was performed [24,25]. Increasing doses of allergen were administrated at 10 min intervals using a spray-device delivering 100 μL per actuation. One puff was administered into each nostril resulting in effective doses of 100, 300, 1.000, and 3.000 SQ units per nasal cavity (Aquagen, ALK-Abelló, Horsholm, Denmark). This scheme was followed until the subject responded with at least 5 sneezes or recorded a symptom score of 2 or more on a scale from 0 to 3 for either nasal secretion or nasal blockage. The dose that produced this effect was chosen for the allergen challenge series and was given once daily for seven days. The allergen challenge was administered two to five hours after the morning dose of the study drug.

### Clinical evaluation

The subjects scored nasal symptoms every morning and evening, starting three days prior to each treatment and continuing until day six after the last treatment day. The scores were entered into diary cards and each registration reflected the preceding twelve hours. Nasal secretion and blockage, respectively, as well as the most severe of the symptoms sneezing and itching were each scored on a four-grade scale where 0 = no symptoms, 1 = mild symptoms, 2 = moderate symptoms, and 3 = severe symptoms. The scores were added to a daily total nasal symptom score (TNSS), with separate scores for morning and evening observations. Morning and evening scores, respectively, from the 5^th^, 6^th^, and 7^th ^allergen-challenge day where added and divided by three resulting in a mean TNSS based on *three *days' observations (range 0-9). In addition, morning and evening scores, respectively, from the 3^rd ^through 7^th ^allergen-challenge day were added and divided by five resulting in a mean TNSS based on *five *days' observations (range 0-9). Nasal symptoms were also scored immediately prior to and 10 min post allergen challenge: Secretion and blockage were scored as described above, whereas the number of sneezes were counted and transformed into a sneezing score by the investigators: 0 sneezes = 0, 1-4 sneezes = 1, 5-9 sneezes = 2, and 10 or more sneezes = 3. The scores were added to a daily post challenge TNSS, and a mean TNSS for post challenge symptom scores calculated from observations made on the 5^th ^through 7^th ^challenge day as described above. The subjects recorded nasal PIF in the morning, immediately prior to and 10 min post allergen challenge, and in the evening using a flow-meter (Clement-Clarke, Harlow, U.K.) equipped with a facial mask. Mean nasal PIF, for morning observations, observations immediately prior to and 10 min post challenge, and evening observations, respectively, from the last three days of each allergen challenge series were calculated.

### Nasal lavages and bradykinin challenges

A 2.5 min nasal lavage with isotonic saline was carried out at the first study visit prior to any treatment and prior to any allergen challenge (*baseline observation*). Nasal lavages were also obtained twice on day seven of each allergen challenge series. Accordingly, a 2.5 min saline lavage was carried out first and this lavage was separated from the allergen challenge on this particular day by at least 1 hour. It was followed by two 30 sec lavages carried out in order to remove remaining solutes on the nasal mucosal surface. (These lavages were discarded.) Fifteen minutes later a nasal spray challenge with bradykinin was performed (in order to improve the recovery of inflammatory mediators [25].) and followed, an additional 15 min later, by a final 2.5 min saline lavage. Nasal lavages were carried out using a pool-device containing 15 ml of isotonic saline [27]. The lavage fluids were kept in the right nasal cavity at all occasions. The recovered lavage fluid was centrifuged and the supernatant was homogenized, prepared in aliquots, and frozen (-30°C). The bradykinin challenge (100 μg) was carried out using a spray-device delivering 100 μL per actuation.

### Analysis

α_2_-Macroglobulin was measured using a radioimmunoassay sensitive to 7.8 ng/mL. The intra- and inter-assay coefficients of variation are between 3.8-6.0% and 3.1-7.2%, respectively [25]. ECP was measured using a fluoroimmunoassay (Pharmacia-Diagnostics, Uppsala, Sweden) with a sensitivity of 2.0 ng/mL. Tryptase was measured using a radioimmunoassay with detection limit of 0.5 ng/mL (Pharmacia-Diagnostics, Uppsala, Sweden). α_2_-Macroglobulin and ECP were analyzed in the lavage fluids as they were, whereas lavage fluids samples were concentrated five times before the analysis of tryptase.

### Statistics

All hypothesis-testing was done using two-sided alternatives at a 5% significance level. The comparison between AZD3778 and placebo was primary and comparisons versus loratadine secondary. Accordingly, no adjustments for multiplicity were applied. The full analysis set was used for all analyses, i.e., all subjects with evaluable data collected from at least two treatment periods. In total 37 of the 38 randomized subjects could be evaluated for efficacy. Change in period means from baseline (i.e., mean over the last three days of the run-in/wash-out period that preceded the treatment period) to treatment in symptom scores and nasal PIF recorded in the diary were compared between treatment groups using an analysis of variance model with subject, period, and treatment as fixed factors and using baseline as a covariate. Period means for symptoms and nasal PIF recorded post-challenge, and variables from the nasal lavages, were compared between treatment groups using analysis of variance models with subject, period and treatment as fixed factors.

## Results

### Pharmacokinetic observations

Plasma levels of AZD3778 were stable late into the treatment series (Table 1). The minimum level recorded was 0.3 μmol/L and a total of six pre-dose samples were below the 3 × A_2 _level of 0.6 μmol/L over treatment days 8, 9, and 10. The median trough plasma levels of AZD3778 for days 8, 9, and 10 were 3.41, 2.79, and 2.95 μmol/L, respectively.

**Table 1:** Summary statistics on plasma concentrations of AZD3778 (μmol/L).

Variable	n	Gmean	CV	Min	Median	Max
Day 1, post-dose	34	5.49	38.5	2.45	5.83	10.40

Day 8, pre-dose	24	2.51	98.0	0.29	3.41	6.27

Day 8, post dose	34	7.77	44.4	3.14	7.95	20.10

Day 9, pre-dose	26	2.21	106.4	0.32	2.79	8.13

Day 9, post dose	34	7.55	36.0	3.15	7.51	15.60

Day 10, pre-dose	34	2.53	71.0	0.28	2.95	7.84

Day 10, post dose	34	7.19	36.5	3.29	7.34	13.50

Plasma levels of AZD3778 were stable during the last three days of the treatment series.

### Post challenge symptom scores

During the last *three days *of the allergen challenge series, AZD3778 and loratadine reduced post-challenge mean TNSS (p < 0.001 and 0.001, respectively) (Tables 2 and 3). In addition, AZD3778, but not loratadine, reduced post-challenge nasal blockage (p = 0.008, c.f. placebo): mean scores for nasal blockage was 1.41, 1.52, and 1.65 for AZD3778, loratadine, and placebo, respectively.

**Table 2:** Period means and ranges for pre- and 10 min post-allergen challenge mean TNSS and mean nasal PIF over the last *three days *of the allergen challenge series.

			Pre challenge		10 min post challenge	
**Variable**	**Treatment**	**n**	**Mean**	**Range**	**Mean**	**Range**

TNSS	AZD3778	35	1.25	0, 3	4.11	0.7, 7.7
	
	Loratadine	35	1.64	0, 8	3.85	1, 8
	
	Placebo	34	1.56	0, 5.5	5.57	1.7, 9

Nasal PIF	AZD3778	35	179	76.7, 247	125	50, 200
	
	Loratadine	35	165	50, 243	114	30, 210
	
	Placebo	34	177	100, 257	108	57, 177

**Table 3:** Treatment comparisons for 10 min post challenge mean TNSS and mean nasal PIF over the last *three days *of the allergen challenge series.

Variable	Treatment	**Mean difference**^**a**^	95% CI	P-value
TNSS	AZD3778 vs. placebo	-1.56	-2.03, -1.09	*<0.001*
	
	Loratadine vs. placebo	-1.87	-2.34, -1.4	*<0.001*
	
	AZD3778 vs. loratadine	0.312	-0.149, 0.773	0.181

Nasal PIF	AZD3778 vs. placebo	16.8	5.73, 27.9	*0.004*
	
	Loratadine vs. placebo	8.67	-2.43, 19.8	0.124
	
	AZD3778 vs. loratadine	8.14	-2.83, 19.1	0.143

^a^Adjusted mean difference from ANOVA.

AZD3778 as well as loratadine reduced post challenge TNSS. In addition, AZD3778 improved post challenge nasal PIF.

### Morning and evening symptom scores

The allergen challenge-induced change in mean TNSS from baseline was lowest in the AZD3778 group during last *three days *of the allergen challenge series, but treatment comparisons did not reach statistically significant differences for either AZD3778 or loratadine compared with placebo: mean TNSS in the morning were 1.66, 1.98, and 2.00 for AZD3778, loratadine, and placebo, respectively. Corresponding values for TNSS in the evening were 1.59, 1.86, and 1.72. Similarly, there were no differences between the treatments regarding morning and evening nasal PIF (data not shown).

In contrast to the three-day observation period, mean TNSS of the last *five days *of the allergen challenge series showed statistically significant reductions in mean TNSS differences between AZD3778 and placebo for morning as well as evening observations (p = 0.023 and 0.022, respectively) (Tables 4 and 5). In contrast, no such effects were observed for loratadine.

**Table 4:** Period means and ranges for TNSS from the diary cards: Period means over *five *days.

			Baseline period		Treatment period	
**Variable**	**Treatment**	**n**	**Mean**	**Range**	**Mean**	**Range**

TNSS -morning	AZD3778	35	0.581	0, 2	1.6	0.2, 4.67
	
	Loratadine	35	0.829	0, 4	1.88	0, 7.2
	
	Placebo	35	0.695	0, 3.33	2	0, 8

TNSS -evening	AZD3778	35	0.486	0, 2	1.48	0, 4.33
	
	Loratadine	35	0.743	0, 4	1.85	0, 8.6
	
	Placebo	34	0.657	0, 3.33	1.88	0, 5.6

**Table 5:** Treatment comparisons for TNSS from the diary cards: Period means over *five *days.

Variable	Treatment	**Mean difference**^**a**^	95% CI	P-value
TNSS -morning	AZD3778 vs. placebo	-0.459	-0.85, -0.067	*0.023*
	
	Loratadine vs. placebo	-0.174	-0.569, 0.22	0.381
	
	AZD3778 vs. loratadine	-0.285	-0.685, 0.115	0.159

TNSS -evening	AZD3778 vs. placebo	-0.562	-1.04, -0.083	*0.022*
	
	Loratadine vs. placebo	-0.172	-0.649, 0.305	0.474
	
	AZD3778 vs. loratadine	-0.39	-0.868, 0.088	0.108

AZD3778 reduced TNSS in the morning as well as in the evening.

^a^Adjusted mean difference from ANOVA.

### Lavage fluid observations

**Figure 1:**
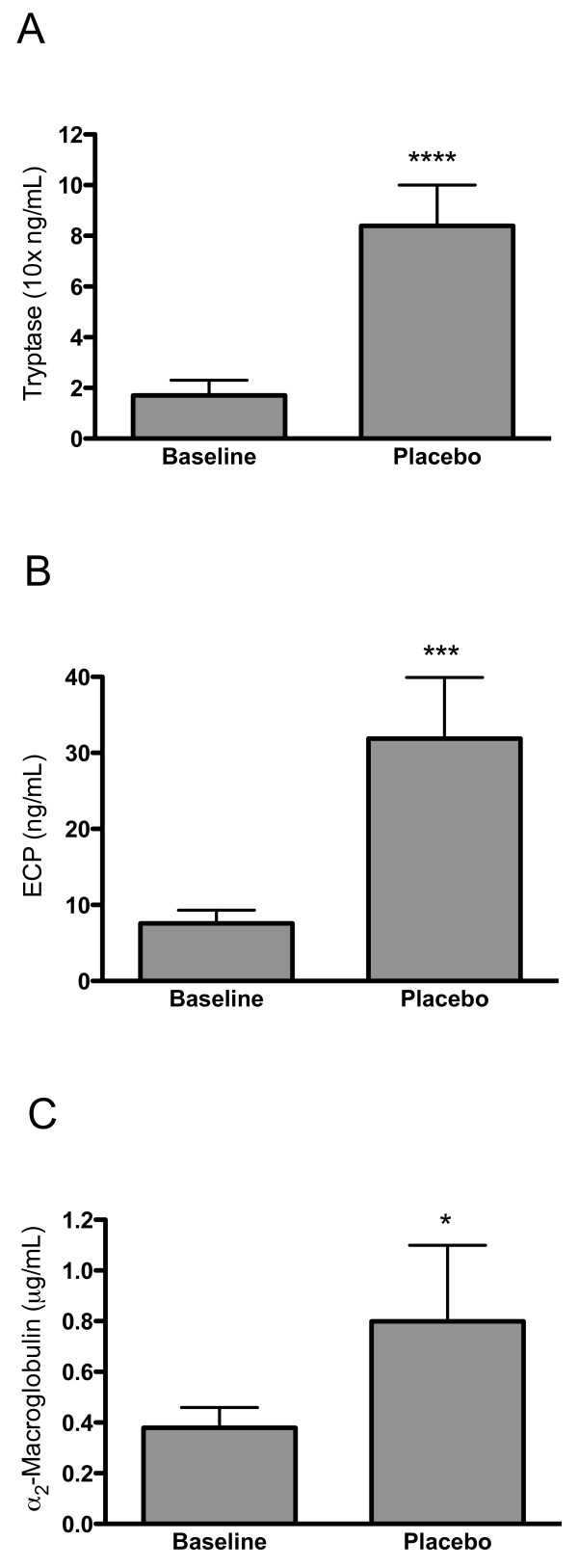
**A. Tryptase (A), ECP (B), and α_2_-macroglobulin (C) in lavages obtained at baseline (before allergen challenge) and in corresponding lavages late into the allergen challenge series (prior to bradykinin challenge) in the placebo run (median values with interquartile ranges)**. The allergen challenge series produced an inflammatory response characterized by increased mast cell and eosinophil activity as well as by plasma exudation. (*Denotes p < 0.05, ***denotes p < 0.001, and ****denotes p < 0.0001.)

**Figure 2:**
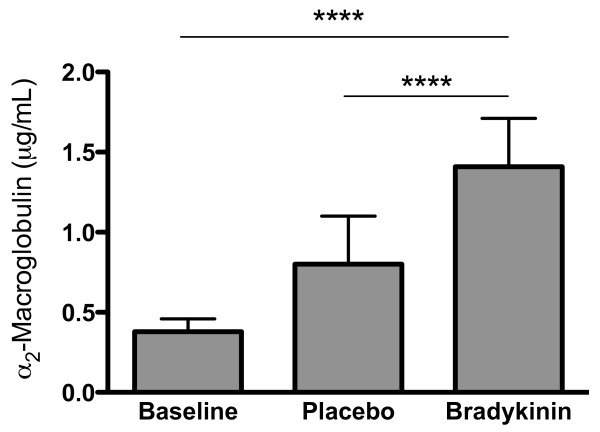
**α_2_-Macroglobulin in lavages obtained at baseline and late into the allergen challenge series prior to and after bradykinin challenge in the placebo run (median values with interquartile ranges)**. Bradykinin produced plasma exudation, a process that may facilitate luminal entry of tissue solutes including tryptase and ECP. (****Denotes p < 0.0001.)

In the placebo run, nasal lavage fluid levels of tryptase, ECP, and α_2_-macroglobulin were elevated in saline lavage fluids collected late into the allergen challenge series (*c.f*. baseline observation prior to the challenge series) (Figure 1). Also, compared with the baseline as well as with the saline lavage carried out late into the allergen challenge series, bradykinin produced increased levels of α_2_-macroglobulin (Figure 2).

**Figure 3:**
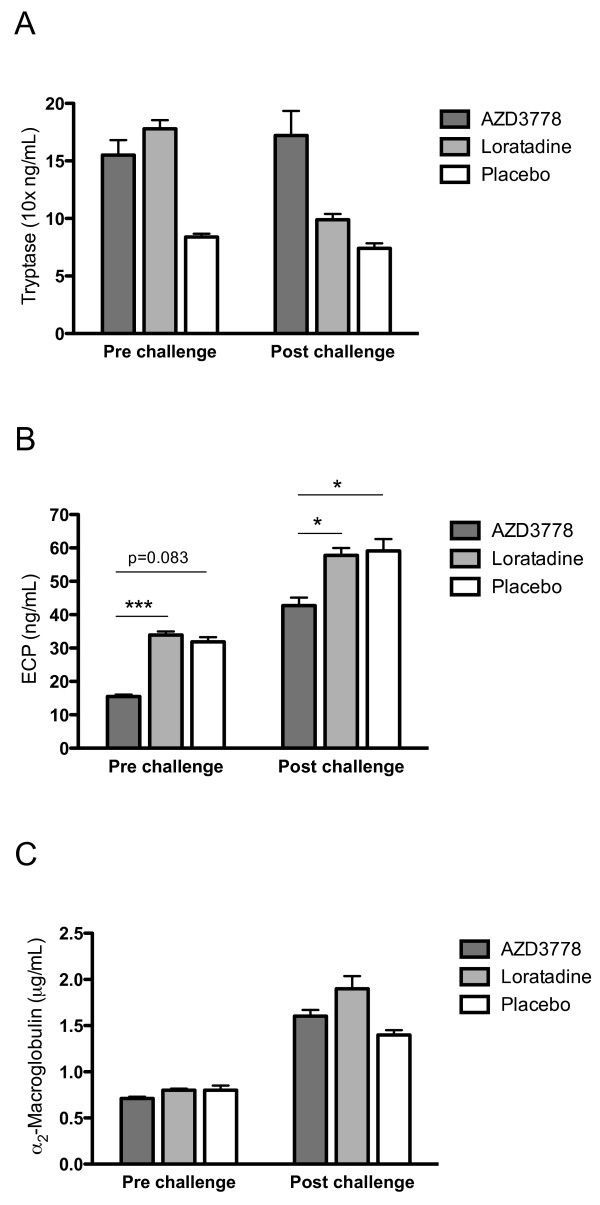
**Tryptase (A), ECP (B), and α_2_-macroglobulin (C) in lavages obtained late into the allergen challenge series prior to and after bradykinin challenge (median values with interquartile ranges)**. AZD3778 reduced the levels of ECP in lavages obtained prior to as well as after bradykinin challenge (*c.f*. placebo). Whereas this change reached borderline statistical significance prior to bradykinin challenge (p = 0.08), it was statistically significant after the challenge. In contrast to AZD3778, loratadine failed to reduce the levels of ECP (*c.f*. placebo). Furthermore, ECP recorded at AZD3778 treatment were significantly lower compared to at treatment with loratadine. The treatments all failed to reduce the lavage fluid levels of tryptase and α_2_-macroglobulin (*Denotes p < 0.05 and ***denotes p < 0.001.)

Whereas loratadine failed to affect the lavage fluid indices that were increased by the repeated allergen challenges, AZD3778 reduced the levels of ECP compared with placebo (Figure 3). For the lavage obtained pre-bradykinin challenge, the numerical reduction in ECP failed to reach statistical significance (Figure 3), whereas the reduction was statistically significant for the post-bradykinin observation (p = 0.038) (Figure 3). Also, focusing on post-bradykinin observations, AZD3778 reduced the levels of ECP compared with loratadine (p = 0.012) (Figure 3). Neither AZD3778 nor loratadine reduced the levels of tryptase and α_2_-macroglobulin (Figure 3).

## Discussion

The present study demonstrates that a dual CCR3 and histamine H_1_-receptor antagonist (AZD3778) can exert anti-eosinophil and symptom-reducing effects in a condition in man characterised by allergic airway inflammation. Our data are of interest with regard to the pharmacology of CCR3-antagonism, the relative importance of eosinophil actions to the symptomatology of allergic rhinitis, and the potential use of AZD3778 in the treatment of allergic rhinitis.

Preceding *in vitro *studies performed by MDS Pharma Services indicted that AZD3778 had a whole blood A_2 _at the CCR3-receptor of 0.2 μmol/L. Furthermore, with regard to antagonism of the histamine H_1_-receptor, a whole blood exposure of 1.0 μmol/L was equivalent to A_2_. In the present study, plasma levels of AZD3778 were stable late into the treatment series. At trough, the plasma concentration of AZD3778 suggested that exposures were close to those required for 24-hour antagonism of the CCR3 and H_1_-receptors.

It is difficult to compare treatment-effects in allergic rhinitis during the pollen season, reflecting uncertainties regarding onset and intensity of natural allergen exposure. Accordingly, it is impossible to perform accurate studies of crossover design and when resorting to parallel group studies these are hampered by inter-individual differences in allergen sensitivity. In order to overcome these problems, we have introduced a model where nasal challenges with individualized doses of allergen are given for seven consecutive days to create a repeatable artificial pollen season characterized by development of rhinitis symptoms and allergic inflammation [24,25]. In the present study, this model was used and around-the-clock rhinitis symptoms were produced by the allergen-challenges in the placebo-run. Notably, symptom scores reached at placebo treatment were very similar to those recorded previously in the model.

Using the mean over the last three days of the allergen challenge series, based on previous evaluations in the present model in which symptoms gradually increased over time [24,25], AZD3778 reduced post-challenge TNSS compared with placebo. In contrast, no statistically significant effects were observed for morning and evening symptoms when focusing on this period. By these characteristics, which were of the same profile and magnitude as for anti-histamines: loratadine (this study) and cetirizine [28], it might be suggested that AZD3778 acted through H_1_-antagonism or that CCR3-antagonism resulted in the same effect-profile as that of anti-histamines. However, AZD3778 also reduced post-challenge nasal blockage and improved post-challenge nasal PIF. Moreover, when focusing on the last five days of the allergen challenge series (which was valid since an effect plateau was reached already at allergen challenge day three), AZD3778 exerted statistically significant reducing effects also on morning and evening symptoms. These latter effects, which were not observed for loratadine, also suggest that CCR3-antagonism *per se *has specific anti-rhinitis effects. Accordingly, CCR3-antagonists may have a potential as a treatment for allergic rhinitis. From a therapeutic point of view, the possibility that AZD3778 exerts CCR3 as well as H_1_-antagonistic effects is attractive. However, compared with previous observations in the present model [24,25], the effect of AZD3778 will likely be inferior that of a topical corticosteroid. Further studies are warranted to evaluate AZD3778 and other CCR3-antagonists in allergic rhinitis.

In the present placebo run, nasal lavage fluid levels of ECP, tryptase, and α_2_-macroglobulin increased during the allergen challenge series compared with baseline data. This was in agreement with previous observations in the present model and indicated a development of allergic airway inflammation [25]. Whereas loratadine failed to affect these indices, AZD3778 reduced the levels of ECP compared with placebo. For the pre-bradykinin challenge lavage, the numerical 37% reduction in ECP failed to reach statistical significance, whereas the reduction was significant for the post-bradykinin observation. (Plasma exudation events acutely produced by histamine is known to improve the recovery of tryptase and ECP [25]: bradykinin was employed in the present study to achieve this and its exudative effect was confirmed.) The effect of AZD3778 likely reflected CCR3-antagonism and not H_1_-antagonism, since it was not observed for loratadine. In contrast to its effects on ECP, AZD3778 failed to affect the levels of tryptase, which disagreed with the observation by Nakamura *et al*. on mast cell stabilizing effects of a CCR3-antagonist in a mouse model [20]. Differences between test systems, species, and the employed antagonists might account for the discrepancy. AZD3778 as well as loratadine also failed to reduce the allergen challenge induced plasma exudation (α_2_-macroglobulin). Taken together, our findings suggest that AZD3778 exerts a selective anti-eosinophil effect. The observation is in keeping with previous findings on such effects by CCR3 antagonism in experimental models [9-12,17-23], and extends them to include allergic rhinitis. While reducing eosinophil activity in allergic rhinitis, the effect profile of AZD3778 is different from what is expected for a topical corticosteroid, which reduces all the employed lavage fluid indices in the present model [25].

In the present study, AZD3778 exerted a moderate anti-eosinophilic and symptom-reducing effect in allergic rhinitis. We might not conclude that the effect on nasal symptoms was secondary to anti-eosinophilic actions associated with CCR3-antagonism. However, the findings suggest that part of the symptomathology of allergic rhinitis depends on eosinophil activity. Indirectly, this is in agreement with observations on seasonal allergic rhinitis as a condition featuring particularly intense eosinophil activity. For instance, eosinophil degranulation (assessed by transmission electron microscopy) is much greater in on-going allergic rhinitis than in asthma [29]. Based on the present findings, and observations by Erjefält et al. [29], it can be suggested that interventions aiming at reducing eosinophil activity in airway inflammation may better be evaluated in allergic rhinitis than in asthma. Such intervention may include IL-5 receptor antagonists, despite the fact that trials with IL-5 active drugs (in asthma) have been disappointing [30-32]. A specific area in which it may be of interest to explore effects of anti-eosinophil active drugs, notably CCR3-antagonists, is the interaction between allergic/eosinophilic inflammation and the common cold. For example, a series of observations indicate the importance of rhinovirus infections to exacerbations of asthma [33], and experimental inoculation studies suggest that CC-chemokines and eosinophils may be involved in this interaction [34,35].

## Conclusion

In summary, we conclude that AZD3778 exerts moderately anti-eosinophilic and symptom-reducing effects in allergic rhinitis. This effect can in part be attributed to CCR3-antagonism.

## List of abbreviations used

BMI: Body mass index; CCR3: CC-chemokine receptor-3; ECP: Eosinophil cationic protein; GM-CSF: Granulocyte-macrophage colony-stimulating factor; IL: Interleukin; MCP-4: Monocyte chemoactive protein-4; PIF: Peak inspiratory flow; RANTES: Regulated on Activation, Normal T-cell Expressed, and Secreted; SQ-unit: Standard quantity unit; TNSS: Total nasal symptoms score.

## Competing interests

Lennart Greiff has consulting agreements with AstraZeneca, CC10 Sweden, and Orexo. Jonas Erjefält has consulting agreements with AstraZeneca, GSK, and Orexo. Morgan Andersson has a consulting agreement with ALK-Abelló. Ash Bahl, Thomas Bengtsson, and Kerstin Dahlström are employees of AstraZeneca. Cecilia Ahlström-Emanuelsson and Henrik Widegren have no relationships to declare. The study was supported in parts by grants from the Swedish Research Council, Skåne County Council, Lund University, and AstraZeneca.

## Authors' contributions

All authors participated in the design of the study. LG, CAE, HW, and MA carried out the clinical experiments. LG and JE were responsible for the analyses of the biological samples. TB performed the statistical analysis. All authors were involved in the interpretation of the results. LG, AB, and MA drafted the manuscript. All authors read and approved the manuscript.
